# Pneumonia

**Published:** 1902-11-22

**Authors:** 


### PNEUMONIA.

After the prolonged spell of fine and open weather which we have lately been enjoying, it is to be feared that the wintry cold, coming suddenly upon us, will prove disastrous to many, and that diseases of the lungs will exact a heavy tribute. It is then peculiarly seasonable and appropriate that the attention of the profession should be directed to the subject of pneumonia as it has been by the delivery, before the Royal College of Physicians, of the Croonian Lectures on "The Natural History and Pathology of Pneumonia"—lectures prepared by the late Dr. Washbourn, but read by Dr. Hale White—and by other papers on pneumonia by various authors which have recently appeared. Among the latter we would specially refer to a clinical lecture by Sir Dyce Duckworth on "The Treatment of Pneumonia" (Brit. Med. Journ., Nov. 15) for we can hardly fail to see that the many brilliant discoveries which have of late been made in regard to the bacteriology of the disease have tended to overshadow and to some extent to put in the background what, after all, ought to be to the practitioner the main point of interest, namely, the treatment of the patient.

The natural history of pneumonia, says Sir Dyce Duckworth, shows that it is an acute disease, that it is truly an inflammation of the lung, but that it is something more. Although it is a local inflammation it is a specific fever due to the influence of a toxin which is generated by two or three specific microbes which evidently are at the bottom of the whole process. The early symptoms of pneumonia consist of a feeling of weariness, a bad frontal headache, and shivering, with which the disease often starts. The patient is too ill for his work and goes to bed, but there may be nothing yet which points to inflammation of the lungs. The experienced eye will, however, detect symptoms worthy of note even at that early stage. A flushed face, rapid breathing, a quick pulse, a high temperature, in the absence of other things which cannot be detected, should certainly make one think of pneumonia in such cases; so one must examine the patient day after day until something definite is found. The signs which indicate a gradual progressive consolidation of the lung are an insidious increase in the frequency of breathing, and a high state of fever, the pulse and respiration reaching usually the proportion of one to two. Then there is the short cough and the expectoration of blood-stained mucus or rusty sputa, sticky and tenacious. When that is present the diagnosis is practically certain. On the third or fourth day there is apt to come out an eruption of herpes on the face, nose, cheek, or chin, which in the majority of cases is a favourable symptom.

Now as to treatment, the late Sir William Gull used to say that the essential thing for a pneumonia patient was to put him in a warm bed, and there is no doubt that a warm bed and good nursing are essential parts of the treatment. Such patients have no appetite, and there is no object in forcing food. They are likely to do very well on a fever diet consisting mainly of milk and beef-tea. The high temperature of 102° to 103° appears to be a necessary part of the disease and one way out of it, so that as long as it keeps within limits one takes no steps to check it. But if the temperature should approach what may be called hyperpyrexia, the limit of which one may place at 105° Fahr., means must be taken to reduce it. This is best done by sponging with ice-water and placing a cradle under the bed covering. If this is not enough one may suspend small buckets of ice from the cradle, and so keep the patient in a cooling atmosphere; or one may apply Leiter's tubes, with ice-water, to the head; or put ice in bags in the axillæ or between the legs. If such means fail one may use some drug, not such things as antipyrin, antifebrin, and phenacetin, which are apt to be mischievous, but quinine in doses of 5 grains every two to four hours, until the temperature gives way. To place the patient in cold baths, as is still largely done in Germany and America, is a severe and exhausting process, which is unnecessary. As to other internal remedies it is usual to prescribe salines, as potassium citrate and ammonium citrate, which are certainly helpful. Quinine is also useful even in the absence of hyperpyrexia, or compound tincture of bark may be added to the saline.

The natural history of the disease shows that the inflammatory process comes to an end about the sixth or seventh day, although the physical signs of consolidation still remain, absorption of the inflammatory products taking some time. Supposing, however, the temperature does not fall by crisis on the seventh day, but goes on to the eighth or even the twelfth day: in such case there is probably some further disorder or complication; either the patient is in a very low condition and the disease is not resolving properly, or there is some pleural effusion, probably empyema, the presence of which is to be ascertained by the insertion of a needle into the dull area.

There are two symptoms connected with the nervous system which are of importance, delirium and insomnia. Delirium is especially apt to occur in pneumonia of the apex. It also occurs in elderly patients and in those who have been intemperate in alcohol. Pneumonia in the drunkard is almost certainly a deadly disease, while double pneumonia in such a patient is absolutely fatal; there is no chance for him.

When insomnia occurs, especially towards the crisis, about the sixth or seventh day, it is a very grave matter, and the question of the use of opium is a very serious one, one also on which there is great difference of opinion. It does not do to be too dogmatic, but there are certainly some cases in which it is beneficial when there is no reason to suppose that the kidneys are affected. A very good plan is to give morphine in small doses with compound spirits of ether, say 15 minims of liquor morphine with 1 fluid drachm of compound spirits of ether. If that is not sufficient the dose may be repeated.

Failure of the heart in pneumonia is a very serious condition, and one which is very apt to occur. The circulation through the lung is blocked, the right heart is distended, and the venous system is engorged. This is a very dangerous condition of affairs; not many hearts can bear the strain for long, and if the organ is weak, owing either to age or degeneration, the strain will prove too much and the patient will die. Under such circumstances blood-letting to the extent of 8 to 12 ozs. may be very useful. Blood-letting again, by way of the application of two or three leeches, is useful in the treatment of the painful dry pleurisy which is an almost constant accompaniment of pneumonia.

If the heart begins to falter and the pulse becomes irregular and small, one should give oxygen, the use of which should not be put off until too late; or strychnine may be injected hypodermically; or musk may be used. In regard to the latter there is only one objection, namely, its costliness. But when musk is really wanted it is well worth the money. " The old physicians used 9 grains to 12 grains of musk made up in the form of a mixture with syrup and mucilage, and I cannot doubt," says Sir Dyce Duckworth, " that they got better results than we do with the small doses." The modern plan is to give a grain or two at a time, generally with spirits of ether.

As to stimulants, many cases in the young are best treated without any at all. But if there be engorgement of the heart, a high temperature, a rapid pulse, the first sound of the heart being barely audible at the aortic area, stimulants should be given. In the majority of cases 2 ozs. to 4 ozs. of brandy are sufficient in the course of each day, but even 12 ozs. may be required. Plenty of pure water to drink should be given in pneumonia as in all febrile conditions. The patient is not to be kept in a hot steam-laden atmosphere.

